# Effects of a very high saturated fat diet on LDL particles in adults with atherogenic dyslipidemia: A randomized controlled trial

**DOI:** 10.1371/journal.pone.0170664

**Published:** 2017-02-06

**Authors:** Sally Chiu, Paul T. Williams, Ronald M. Krauss

**Affiliations:** Children’s Hospital Oakland Research Institute, Oakland, California, United States of America; Universita degli Studi di Milano, ITALY

## Abstract

**Background:**

Previous studies have shown that increases in LDL-cholesterol resulting from substitution of dietary saturated fat for carbohydrate or unsaturated fat are due primarily to increases in large cholesterol-enriched LDL, with minimal changes in small, dense LDL particles and apolipoprotein B. However, individuals can differ by their LDL particle distribution, and it is possible that this may influence LDL subclass response.

**Objective:**

The objective of this study was to test whether the reported effects of saturated fat apply to individuals with atherogenic dyslipidemia as characterized by a preponderance of small LDL particles (LDL phenotype B).

**Methods:**

Fifty-three phenotype B men and postmenopausal women consumed a baseline diet (55%E carbohydrate, 15%E protein, 30%E fat, 8%E saturated fat) for 3 weeks, after which they were randomized to either a moderate carbohydrate, very high saturated fat diet (HSF; 39%E carbohydrate, 25%E protein, 36%E fat, 18%E saturated fat) or low saturated fat diet (LSF; 37%E carbohydrate, 25%E protein, 37%E fat, 9%E saturated fat) for 3 weeks.

**Results:**

Compared to the LSF diet, consumption of the HSF diet resulted in significantly greater increases from baseline (% change; 95% CI) in plasma concentrations of apolipoprotein B (HSF vs. LSF: 9.5; 3.6 to 15.7 vs. -6.8; -11.7 to -1.76; p = 0.0003) and medium (8.8; -1.3 to 20.0 vs. -7.3; -15.7 to 2.0; p = 0.03), small (6.1; -10.3 to 25.6 vs. -20.8; -32.8 to -6.7; p = 0.02), and total LDL (3.6; -3.2 to 11.0 vs. -7.9; -13.9 to -1.5; p = 0.03) particles, with no differences in change of large and very small LDL concentrations. As expected, total-cholesterol (11.0; 6.5 to 15.7 vs. -5.7; -9.4 to -1.8; p<0.0001) and LDL-cholesterol (16.7; 7.9 to 26.2 vs. -8.7; -15.4 to -1.4; p = 0.0001) also increased with increased saturated fat intake.

**Conclusions:**

Because medium and small LDL particles are more highly associated with cardiovascular disease than are larger LDL, the present results suggest that very high saturated fat intake may increase cardiovascular disease risk in phenotype B individuals. This trial was registered at clinicaltrials.gov (NCT00895141).

**Trial registration:**

Clinicaltrials.gov NCT00895141.

## Introduction

Current dietary guidelines aim at limiting saturated fat intake in large part because of its ability to increase LDL-cholesterol (LDL-C) levels and presumably, cardiovascular disease (CVD) risk. However, several recent meta-analyses and systematic reviews have concluded that saturated fat *per se* is not associated with greater CVD risk [1–3]. This may be due in part to differential effects of saturated fat on LDL subclass concentrations. Small and medium sized LDL particles have been shown to be more strongly associated with CVD outcomes than larger LDL [4–7]. We have previously reported results from a dietary intervention trial indicating that increased intake of total saturated fatty acids, particularly myristic (14:0) and palmitic (16:0) acids, correlated with increased plasma levels of larger LDL particles, but not with change in smaller LDL or apoB concentrations [8]. Moreover, in a subsequent clinical trial, we showed that in the context of reduced carbohydrate intake, the increase in LDL-C resulting from exchange of dietary saturated fat for monounsaturated fat was due primarily to higher concentrations of cholesterol-enriched larger LDL, without changes in smaller LDL or apoB [9]. Saturated fat also raises HDL-cholesterol (HDL-C) [10], which could potentially offset an atherogenic effect of raising LDL-C.

Atherogenic dyslipidemia is the most common dyslipidemia associated with obesity and insulin resistance and is characterized by elevated plasma triglycerides, low HDL-C, and increased levels of small, dense LDL particles [9]. Individuals with an abundance of small, dense LDL particles have been categorized as LDL phenotype B, while individuals with predominantly larger LDL particles have been categorized as LDL phenotype A [11]. The expression of LDL phenotype B is influenced by genetic predisposition, dietary macronutrient intake, and body weight [12]. High carbohydrate intake promotes increased plasma levels of liver-derived triglyceride-rich lipoproteins (VLDL) that can give rise to small, dense LDL particles and a conversion to or exacerbation of phenotype B [12].

The objective of the present study was to determine the effects of high saturated fat intake, primarily from dairy fat, on lipoprotein subclass concentrations in LDL phenotype B individuals. Based on our previous results [9] we hypothesized that the substitution of saturated fat for monounsaturated fat would preferentially increase concentrations of large, but not small, LDL particles, and that this would be accompanied by an increase in larger HDL particles.

## Materials and methods

The trial protocol (S1 Protocol) and CONSORT (S1 CONSORT Checklist) checklist are available as supporting information.

### Study population

Participants were recruited through internet advertisements and from our extensive database of previous study participants. Eligibility was based on the following criteria: male or postmenopausal female ≥ 18 y, non-smoking, LDL phenotype B as determined by ion mobility (see below), BMI 25–35 kg/m^2^, blood pressure < 150/90 mm Hg, fasting blood glucose < 7.0 mmol/L, plasma triglycerides < 5.65 mmol/L, and total-cholesterol (TC) and LDL-C ≤ 95^th^ percentile for age and sex at screening. Participants had no history of diabetes, CVD, or other chronic disease, and were not taking dietary supplements, recreational drugs, drugs known to affect lipid metabolism, blood thinning agents, dietary supplements, or hormones. Premenopausal women were excluded because of their low prevalence of LDL phenotype B [13].

The study was conducted in free-living participants at the Cholesterol Research Center (Berkeley, CA). All participants provided written informed consent. The protocol was reviewed and approved by the Institutional Review Board of the University of California, San Francisco Benioff Children’s Hospital Oakland. The trial is registered at clinicaltrials.gov (NCT00895141).

#### Study design and dietary intervention

The study was conducted between April 2009 and December 2010. Participants consumed a baseline diet for 3 weeks followed by randomization to a low saturated fat (LSF) or high saturated fat (HSF) experimental diet for 3 weeks. They were randomly assigned to one of the two diets in randomly determined blocks of 2, 4, 6, or 8 individuals using a uniform random-number generator by a statistician who was not otherwise involved in recruitment or screening. Diet assignments were kept in sealed envelopes and assigned to the participant by the clinic staff 1–2d before starting the experimental diet. Investigators and staff assessing plasma outcomes were blinded to diet assignment, while clinic staff was not. Participants were not informed of their diet assignment, but due to the nature of the diets, were likely able to identify the experimental diet. Unblinding was performed after all data collection for all participants was completed. During the 3 wk baseline diet and 3 wk experimental diet, participants met with study staff weekly for dietary counseling, to receive study foods, and to be weighed. Body weight was stabilized by adjusting energy intake if needed. At the end of the baseline diet and experimental diet, participants visited the clinic for clinical and laboratory measurements.

Composition of the diets is shown in Table 1. The LSF and HSF diets were designed to have comparable amounts of carbohydrate, protein, and total fat with differences in saturated fat achieved by exchange for monounsaturated fat. Monounsaturated fat was chosen as the exchanged nutrient because this substitution has a smaller effect on LDL-C than does substituting either carbohydrate or polyunsaturated fat [10]. High vs. low/non-fat dairy products were the major sources of differences in saturated fatty acid content. Participants were provided with two standardized entrees and a snack per day and menus, shopping lists, and instructions for preparation of remaining food items. Diets were prescribed using a rotating 4-day menu. Menus were designed and entrees produced by the Bionutrition Unit of the University of California, San Francisco Clinical and Translation Sciences Institute (San Francisco, CA) using Pronutra software. Nutrient content of dairy products and daily menus was analyzed by compositional analysis (Covance Inc, Kalamazoo, MI).

**Table 1:** Composition of baseline and experimental diets^a^.

	Calculated^b^			Analyzed^c^		
	Baseline	LSF	HSF	Baseline	LSF	HSF
Carbohydrate, *%E*	55	35	35	nm	37	39
Protein, %*E*	15	25	25	nm	25	25
Fat, %*E*	30	40	40	nm	37	36
SFA	8	9	21	nm	9	18
MUFA	12	24	11	nm	19	9
PUFA	8	7	6	nm	7	6
Cholesterol, *mg*	219	374	385	nm	281	367

^a^Values given for a 12,557 kJ menu. %E, % energy; MUFA, monounsaturated fatty acids; nm, not measured; PUFA, polyunsaturated fatty acids SFA, saturated fatty acids.

^b^Calculated values include adjustments made after compositional analysis of individual SFA of dairy products and entrées performed during diet development.

^c^Compositional analysis of a complete week of foods and beverages.

### Laboratory measurements

Blood samples were collected after a 12–14 h overnight fast at screening and on two consecutive days after consumption of the baseline diet (day 20 and 21) and the randomized diet (day 41 and 42). Plasma TC, HDL-C, triglycerides (TG), and glucose were measured by enzymatic end-point measurements utilizing enzyme reagent kits on an Express 550 Plus analyzer (Ciba-Corning Diagnostics Corp., Oberlin, OH). TC, HDL-C, and TG were consistently in control as monitored by the Centers for Disease Control and Prevention-National Heart, Lung, and Blood Institute standardization program. LDL-C was calculated using the Friedewald equation [14]. ApoB and apoAI were measured by immunoturbidimetric assays (Express 550 Plus Analyzer: Bacton Assay Systems, San Marcos, CA) [15, 16]. LDL peak particle diameter, LDL phenotype, and lipoprotein particle concentrations were measured by ion mobility, which uses gas-phase differential electrophoretic macromolecular mobility to directly measure lipoprotein particle concentrations [17]. Individuals with LDL peak particle diameter below < 217.5Å (midpoint of intermediate zone) were defined as LDL phenotype B, whereas those with LDL peak particle diameter ≥ 217.5Å were defined as LDL phenotype A [18]. Nondenaturing gradient gel electrophoresis with lipid staining of plasma was performed to confirm LDL ion mobility results as previously described [19]; in this procedure, cholesterol concentration for each LDL subclass was assessed by multiplying LDL cholesterol by the percentage of area under the curve defined for each subclass. Plasma cholesteryl ester transfer protein (CETP) activity was measured using a commercial assay (Roar Biomedical, New York, NY).

For measurements of hepatic lipase (HL) and lipoprotein lipase (LPL) activities, blood was collected after a bolus of intravenous heparin (60U/kg). HL activity was measured by selective inhibition of LPL with protamine sulfate as previously described [20]. LPL activity was calculated as the difference between lipase activities measured with and without protamine sulfate.

### Statistical procedures

Fasting TC, LDL-C, nonHDL-C, TG, HDL-C, and lipoprotein particle concentrations were calculated as the mean of two measurements. Participants’ characteristics at randomization and at the end of the baseline diet are presented as mean ± SD. The primary outcomes were differences in fasting plasma lipid and lipoprotein concentrations from baseline between the LSF and HSF diets. Secondary outcomes were differences in CETP, HL, and LPL activities from baseline between the diets. Analysis of covariance (ANCOVA) was used to test whether the mean 3-wk changes from baseline differed between the LSF and HSF diets when adjusted for sex, age, and baseline BMI. An N of 25 per group in a parallel design was estimated to yield a detectable difference of percent change from baseline between groups at 80% power and 5% significance (two-sided) of 22% for large LDL concentrations, 1.7% for LDL peak diameter, and 25% for large HDL concentrations. Based on previous results [8] we estimated that these differences would be sufficient for detecting the expected effects of the low vs. high saturated fat diets. All p-values for diet-induced differences in 3-wk changes were determined based on differences in log transformed values. Log transformations were used to achieve greater consistency with normally distributed outcome measures. The differences in the logs were converted to percent change in the tables to be more easily interpreted. Spearman’s correlation coefficients (ρ) were used to evaluate the relationships between changes in lipoprotein measurements and enzyme activities. Changes in CETP and HL activity were added to the ANCOVA model described above to test for independent relationships with lipoprotein measurements. A p-value < 0.05 was considered significant. Power calculations and tests of hypotheses were performed using JMP 9.0 (SAS) without adjustment for multiple hypothesis testing.

## Results

### Study participants

**Fig 1:**
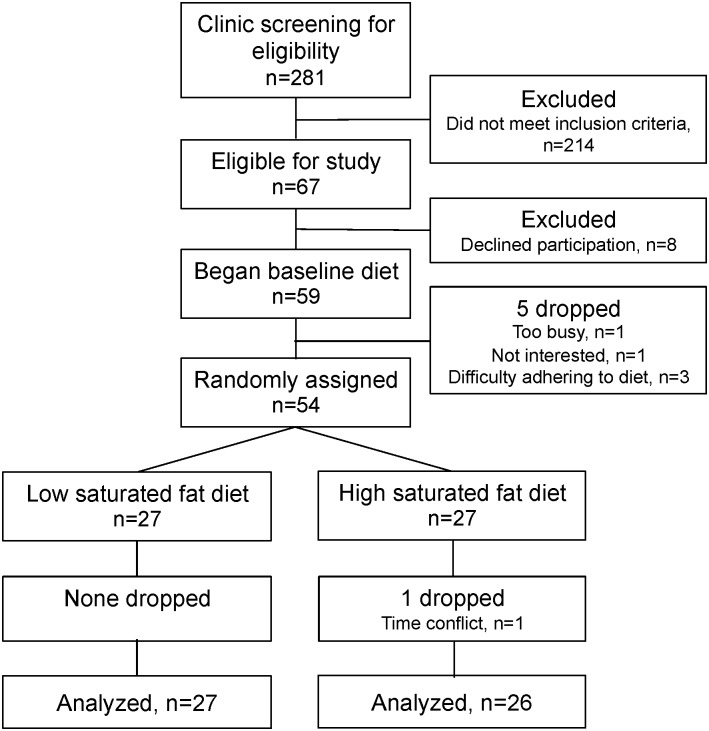
CONSORT flow diagram.

Fig 1 shows the details of participant recruitment and enrollment. Forty-five men and eight postmenopausal women completed the study. Their baseline characteristics are shown in Table 2. Note that despite screening study participants for LDL phenotype B, changes in phenotypes in the period between enrollment and completion of the baseline diet resulted in 11 participants who were phenotype A at randomization; these changes did not differ significantly between the two diet arms. Baseline BMI differed significantly between the LSF and HSF groups and was therefore used as a covariate in analyses of the diet effects. There were no other significant baseline differences between diet groups. There were also no significant changes in body weight during the dietary intervention in either the LSF (-0.3 ± 0.2 kg, p = 0.08) or HSF (0.1 ± 0.1 kg, p = 0.71) groups.

**Table 2:** Participant characteristics at randomization (at the end of the baseline diet)^a^.

	LSF	HSF	p-value^b^
M/F, *N*	21/6	24/2	0.13
Age, *y*	40 ± 12	46 ± 14	0.11
BMI, *kg/m*^*2*^	30.7 ± 2.4	28.9 ± 2.9	0.018
Waist, *cm*	103 ± 7	101 ± 7	0.21
TC, *mmol/L*	4.77 ± 1.01	4.51 ± 0.78	0.27
LDL-C, *mmol/L*	2.75 ± 0.73	2.72 ± 0.73	0.85
HDL-C, *mmol/L*	0.95 ± 0.21	0.93 ± 0.12	0.70
Non-HDL-C, *mmol/L*	3.83 ± 0.88	3.57 ± 0.73	0.26
TG^c^, *mmol/L*	2.46 ± 1.45	1.91 ± 0.97	0.07
ApoB, *g/dl*	9.2 ± 2.0	8.4 ± 1.6	0.11
ApoAI, *g/dl*	10.5 ± 15	10.2 ± 1.2	0.50
LDL phenotype, *N*	4A/23B	7A/19B	0.28
LDL peak diameter, *Å*	213.3 ± 4.2	215.0 ± 4.8	0.16
Total VLDL, *nmol/L*	167.5 ± 46.8	150.6 ± 43.1	0.18
Large VLDL, *nmol/L*	32.8 ± 12.8	27.2 ± 15.1	0.15
Medium VLDL, *nmol/L*	75.6 ± 23.8	67.5 ± 22.1	0.21
Small VLDL, *nmol/L*	59.1 ±15.7	55.9 ± 11.4	0.40
IDL, *nmol/L*	135.9 ± 33.3	137.1 ± 31.8	0.89
Total LDL, *nmol/L*	1256 ± 229	1246 ± 314	0.90
Large LDL, *nmol/L*	444 ± 152	493 ± 173	0.28
Medium LDL, *nmol/L*	252 ± 79	255 ± 99	0.90
Small LDL, *nmol/L*	295 ± 104	262 ± 119	0.29
Very small LDL, *nmol/L*	265 ± 183	236 ± 171	0.56
Total HDL, *nmol/L*	4964 ± 1582	4880 ± 1041	0.82
Large HDL, *nmol/L*	793 ± 391	739 ± 168	0.93
Small HDL, *nmol/L*	4170 ± 1388	4141 ± 990	0.52
HL activity^d^, *umol/ml/hr*	22.7 ± 8.5	20.1 ± 6.9	0.26
LPL activity^d^, *umol/ml/hr*	5.2 ± 3.3	6.3 ± 2.5	0.21
CETP activity, *nmol/ml/hr*	21.7 ± 5.2	21.9 ± 5.2	0.88

^a^Mean ± SD. Apo, apolipoprotein; CETP, cholesteryl ester transfer protein; HL, hepatic lipase; HDL-C, HDL-cholesterol; HSF, high saturated fat diet; IDL, intermediate density lipoprotein; LPL, lipoprotein lipase; LDL-C, LDL cholesterol; LSF, low saturated fat diet; TC, total cholesterol; TG, triglycerides; VLDL, very low density lipoprotein.

^b^Student’s t-test or Chi-squared test.

^c^Log transformed prior to analysis.

^d^N = 20/6 (M/F) for the LSF group and 21/2 (M/F) for the HSF group. Missing data were due to participants who did not complete a postheparin blood draw.

### Plasma lipids and lipoproteins

Compared to the LSF diet, the HSF diet significantly increased plasma TC, LDL-C, and apoB levels and marginally increased plasma TG (Table 3). There were no significant changes in HDL-C or apoAI.

**Table 3:** Percent changes from baseline in plasma lipid and lipoprotein concentrations in men and women with atherogenic dyslipidemia after 3 wk of consuming either a low or high saturated fat diet^a^.

	LSF (N = 26)	HSF (N = 27)	P^b^
ΔTG	-11.5 (-23.4, 2.2)	9.8 (-5.2, 27.2)	0.06
ΔTC	-5.7 (-9.4, -1.8)	11.0 (6.5, 15.7)	<0.0001
ΔLDL-C	-8.7 (-15.4, -1.4)	16.7 (7.9, 26.2)	0.0001
ΔHDL-C	2.8 (-2.0, 7.8)	2.8 (-2.0, 7.9)	0.99
ΔNonHDL-C	-7.9 (-12.3, -3.3)	13.2 (7.7, 19.0)	<0.0001
ΔApoB	-6.8 (-11.7, -1.6)	9.5 (3.6, 15.7)	0.0003
ΔApoAI	-1.3 (-4.8, 2.2)	3.2 (-0.5, 7.1)	0.11

^a^percent change from baseline, mean (95% CI), adjusted for age, gender, and baseline BMI. Apo, apolipoprotein; HDL-C, HDL-cholesterol; LDL-C, LDL cholesterol; TC, total cholesterol; TG, triglycerides.

^b^calculated from one-way ANCOVA using the difference in the log transformed values, adjusted for age, gender, and baseline BMI. Percent changes shown were derived by conversion from the differences in log transformed values.

Total, medium, and small LDL particle concentrations as measured by the ion mobility method were all increased after the HSF diet compared with the LSF diet (Table 4), although the p-values (p = 0.02 to 0.03) were no longer significant after adjustment for multiple testing using the Bonferroni correction. There were no other significant differences in VLDL, intermediate density lipoprotein (IDL), LDL, or HDL subfraction concentrations or LDL peak particle diameter as measured by ion mobility after consuming the LSF vs. the HSF diets (Table 4), and no differences in the proportion of LDL phenotype or LDL phenotype conversions (p = 0.23, data not shown). Small LDL-C measured by gradient gel electrophoresis was significantly higher after the HSF compared to the LSF diet (p<0.05, S1 Fig).

**Table 4:** Percent changes from baseline in plasma lipoprotein subfractions and lipoprotein remodeling enzyme activities in men and women with atherogenic dyslipidemia after 3 wk of consuming either a low or high saturated fat diet^a^.

	% Δ from baseline		
	LSF (N = 26)	HSF (N = 27)	P^b^
ΔTotal VLDL	-7.8 (-16.4, 1.6)	-1.3 (-10.7, 9.0)	0.37
ΔLarge VLDL	-13.3 (-26.3, 1.9)	0.0 (-15.3, 18.1)	0.26
ΔMedium VLDL	-6.9 (-16.3, 3.6)	-1.1 (-11.2, 10.3)	0.46
ΔSmall VLDL	-7.9 (-15.7, 0.7)	-0.7 (-9.3, 8.7)	0.28
ΔIDL	-8.3 (-15.7, -0.4)	3.5 (-5.0, 12.7)	0.06
ΔTotal LDL	-7.9 (-13.9, -1.5)	3.6 (-3.3, 11.0)	0.03
ΔLarge LDL	2.1 (-7.1, 12.2)	8.6 (-1.4, 19.6)	0.40
ΔMedium LDL	-7.3 (-15.7, 2.0)	8.8 (-1.3, 20.0)	0.03
ΔSmall LDL	-20.8 (-32.8, -6.7)	6.1 (-10.3, 25.6)	0.02
ΔVery small LDL	-19.3 (-31.1, -5.3)	-11.8 (-25.0, 3.7)	0.48
ΔLDL peak diameter	0.7 (0.1, 1.3)	0.2 (-0.5, 0.8)	0.27
ΔTotal HDL	0.6 (-9.2, 11.4)	6.3 (-4.3, 17.9)	0.49
ΔLarge HDL	-2.6 (-11.0, 6.7)	0.5 (-8.4, 10.2)	0.66
ΔSmall HDL	1.0 (-9.4, 12.5)	7.1 (-4.1, 19.6)	0.48
ΔHL activity^c^	-9.5 (-15.4, -3.2)	1.3 (-5.8, 8.8)	0.04
ΔLPL activity^c^	-15.7 (-38.2, 15.2)	-8.2 (-33.3, 26.2)	0.72
ΔCETP activity	-0.6 (-7.0, 6.2)	10.0 (2.7, 17.7)	0.05

^a^percent change from baseline, mean (95%CI), adjusted for age, gender, and baseline BMI. CETP, cholesteryl ester transfer protein; HL, hepatic lipase; IDL, intermediate density lipoprotein; LPL, lipoprotein lipase; VLDL, very low density lipoprotein.

^b^calculated from one-way ANCOVA using the difference in the log transformed values, adjusted for age, gender, and baseline BMI. Percent changes shown were derived by conversion from the differences in log transformed values.

In these analyses, gender was included as a covariate and there were no gender by diet interactions for outcomes. When analyses were restricted to males, results for lipid and lipoprotein outcomes remained similar. However, the p-values increased slightly to become non-significant for medium, small, and total LDL concentrations (p = 0.10, 0.06, and p = 0.06, respectively) likely due to reduced sample size.

### Plasma activities of HL, LPL, and CETP

Both plasma HL and CETP activities were higher on the HSF diet compared to the LSF diet, but with borderline statistical significance (p = 0.04 and 0.05, respectively). There was no significant difference in LPL activity (Table 4).

We explored the relationships of changes in CETP and lipase activities with changes in lipid and lipoprotein measurements. There were no significant diet interactions for these associations; thus the data for the two diets were combined for analysis (S1 Table). Changes in HL activity were correlated positively with changes in TG, TC, apoB, non-HDLC, large and intermediate VLDL particles, and small and very small LDL, and negatively with LDL peak particle diameter. LPL activity changes were negatively correlated with changes in TG and large VLDL. Changes in CETP activity were significantly positively correlated with changes in TG, TC, apoB, non-HDLC, large, medium, and small VLDL, IDL, and medium and small LDL.

Finally, we tested whether HL or CETP activities were significant covariates for the effect of increased saturated fat on small and medium LDL particles. In a model including age, gender, baseline BMI, saturated fat level, and changes in CETP and HL activity, changes in CETP activity remained significantly associated with small LDL (p = 0.009) and medium LDL (p = 0.046), while changes in HL activity (p = 0.57 and 0.46, respectively) and saturated fat intake (p = 0.12 and 0.09, respectively) were not.

## Discussion

Recent evidence has shown that although increased intake of saturated fatty acids can raise LDL-C when substituted for either carbohydrate or cis-unsaturated fatty acids [21], the substitution for carbohydrate is not associated with higher CVD risk [22–25]. We hypothesized that the dissociation of LDL-C change from effects on CVD risk may be due in part to a preferential effect of saturated fat on cholesterol-enriched large LDL particles, which are not as strongly associated with CVD risk as are small and medium sized LDL particles [4–6]. In contrast, we and others have shown that higher carbohydrate intake promotes selective increases in levels of small LDL particles [9, 26–28]. In one of these studies, we found that in the context of reduced carbohydrate intake (26% E), increased intake of saturated fatty acids derived primarily from dairy fat preferentially raised large LDL without increasing small LDL or apoB concentrations [9]. This is consistent with results from other intervention studies [8, 29–33] and observational cohort studies [34–36] showing that diets high in saturated fat, derived in many cases from dairy sources, increased peak LDL particle diameter, and/or levels of larger LDL without raising levels of smaller LDL particles. Other studies however, have reported no significant effect of saturated fat on LDL particle size distribution [37–40]. In the present study, we found that with substitution of saturated fatty acids derived primarily from dairy foods for monounsaturated fatty acids, increased LDL-C was associated with increased apoB, and total, small, and medium-sized LDL particle concentrations. There were, however, no significant changes in large LDL, large HDL, or LDL peak diameter. We suggest a number of possible factors that may contribute, singly or in combination, to the discrepancy of these findings from those that we reported previously [9].

First, in the present trial, we enrolled men and postmenopausal women, who on screening expressed the small LDL particle phenotype B, whereas the majority of participants in our earlier study expressed large LDL phenotype A, and there were insufficient numbers of phenotype B individuals to permit testing the possibility of differential responses as a function of baseline LDL particle size phenotypes or other lipid and lipoprotein parameters. We are aware of one other report showing an effect of saturated fat on medium and smaller LDL particles. Gill, et al., showed a stepwise decrease in medium and smaller LDL particles with decreasing saturated fat intake (15%E to 11%E to 7%E) in 35 hypercholesterolemic individuals [41]. Thus is it possible that dyslipidemic individuals may be preferentially susceptible to saturated fat-induced increases in small and medium-sized LDL particles.

Second, the carbohydrate content of the diets in the present study was substantially greater than in our earlier trial (37–39% vs, 26%E). It may be that the effects of higher carbohydrate intake on pathways responsible for increased levels of smaller LDL particles create greater susceptibility to the effects of dietary saturated fat on small LDL particle concentrations. Further studies would be required to confirm such an interaction between dietary carbohydrate and saturated fat.

Finally, the saturated fatty acid content of the high saturated fat diet in the present study was 20% greater than in our previous study (18% vs 15%E), compared in both cases with 9%E. It is therefore possible that there is a threshold above which saturated fat induces metabolic changes resulting in increased levels of smaller LDL particles. Saturated fat is thought to increase LDL-C primarily through down-regulation of hepatic LDL receptor activity, leading to reduced clearance of LDL particles [42, 43]. While smaller LDL particles are less dependent on LDL receptor-mediated uptake than larger LDL [44], it may be that the very high level of saturated fat in the present study suppresses LDL receptor activity sufficiently so as to reduce plasma clearance of smaller LDL in individuals with the small LDL phenotype. It is also conceivable that the high levels of saturated fat studied here might increase direct hepatic secretion of small LDL or their precursors, or perhaps suppress non-LDL receptor pathways responsible for clearance of smaller LDL particles. Consistent with the possibility of a threshold effect of very high dietary saturated fatty acid intake on levels of smaller vs. larger LDL particles is the observation from the Framingham cohort [36] that LDL peak diameter was larger in individuals in the 25–75^th^ percentile of saturated fat intake (mean: 12.5%E), compared to those in either the < 25^th^ percentile (mean: 9%E) or > 75^th^ percentile (mean: 17%E).

We performed exploratory analyses using data from both diet arms to test for relationships of changes in activities of factors involved in lipoprotein catabolism and remodeling with changes in plasma lipids and lipoproteins. As expected, changes in LPL were inversely correlated with changes in TG and TG-rich VLDL particles. Positive correlations of change in hepatic lipase activity with changes in larger VLDL particles and smaller LDL particles, are consistent with previous reports [45, 46] and with the role of hepatic lipase in generating small, dense LDL [47]. A novel finding in the present study was the positive correlation of changes in CETP activity with changes in all of the apoB-containing lipoprotein particle fractions with the exception of large and very small LDL.

Although mean changes in both HL and CETP from the baseline diet showed only marginally significant differences between the HSF and LSF diets, inclusion of both of these activities in a model also containing diet assignment indicated that change in CETP but not HL activity remained significantly associated with change in small LDL particle concentration, suggesting that the increase in CETP may have contributed in part to the effect of high saturated fat intake on these particles, and that the HL association was dependent on this CETP effect. This possibility is consistent with previous studies demonstrating increases in CETP mass or activity with very high saturated fat intakes (≥19%E) [48–50], and positive correlations between changes in CETP activity and changes in total- and LDL-cholesterol [48, 49]. Moreover, in monkeys fed a high saturated fat diet, increased CETP activity was associated with reduced LDL receptor activity [51].

Strengths of our study include a lack of confounding by weight loss, detailed lipoprotein subclass measurements using two different analytical methods, and a saturated fat-induced increase in LDL-C consistent with predictive formulas [21] (data not shown), indicative of good dietary adherence. One limitation of the study is that since all participants were LDL phenotype B when enrolled, we are unable to determine whether the results are specific to individuals with this metabolic trait. Although some individuals converted to LDL phenotype A after the baseline diet, there were insufficient numbers to formally evaluate potential differences in response between the two baseline LDL phenotypes. In addition, the dietary intervention was short-term and may not reflect long-term intake of high saturated fat diets. Finally, the increase in saturated fat content on the HSF diet was primarily derived from dairy foods, and thus the findings may not be applicable to other sources of saturated fat.

In conclusion, we found that the increase in LDL-C resulting from very high saturated fat intake in individuals with a preponderance of small LDL was associated with an increase in apoB, and total, medium-sized, and small LDL particles. These results, in conjunction with previous studies, suggest that saturated fat may have heterogeneous effects on levels of atherogenic LDL particles that may depend on the amount of saturated fat consumed, the dietary context, particularly concomitant carbohydrate intake, and/or predisposition to atherogenic dyslipidemia.

## Supporting information

S1 CONSORT Checklist CONSORT checklist. (PDF)

S1 Dataset Minimal data set for results presented. (XLSX)

S1 Fig Changes in LDL subfractions as measured by gradient gel electrophoresis after 3 wk of consuming either a low saturated fat (LSF, N = 26) or high saturated fat (HSF, N = 27) diet. Concentration of each LDL subclass was determined by multiplying the LDL cholesterol with the percentage of area under the curve defined for each subclass. *Different from LSF diet, p <0.05.
(TIF)

S1 Protocol Trial protocol. (PDF)

S1 Table Spearman’s rho correlations of change in enzyme activities and change in lipid and lipoproteins. (PDF)
