# Supplementary figures and images for: Effects of a very high saturated fat diet on LDL particles in adults with atherogenic dyslipidemia: A randomized controlled trial
Source: PLoS One. 2017 Feb 6;12(2):e0170664. doi: 10.1371/journal.pone.0170664 (PMC5293238; doi:10.1371/journal.pone.0170664)

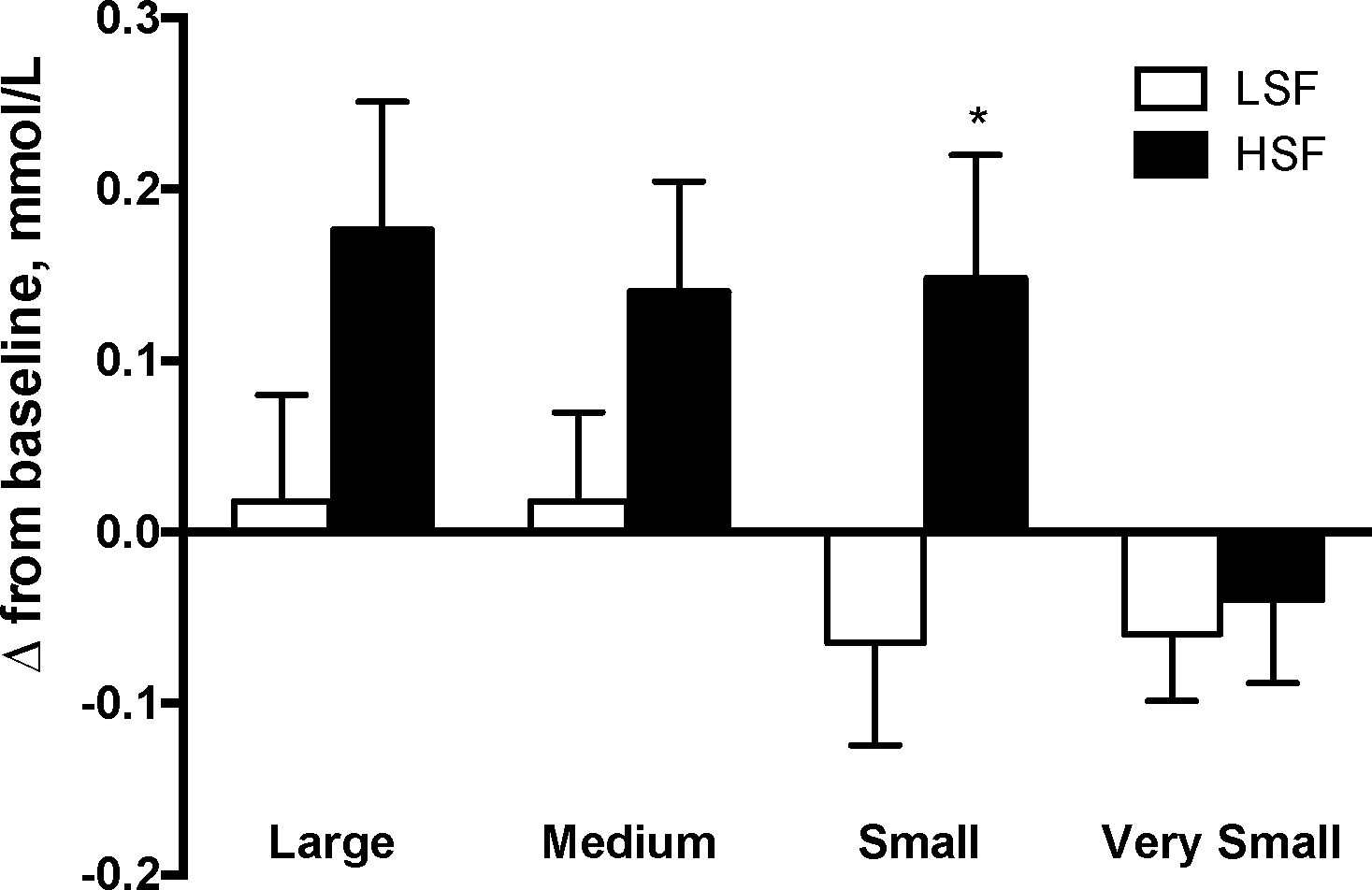

Supplement: S1 Fig — Concentration of each LDL subclass was determined by multiplying the LDL cholesterol with the percentage of area under the curve defined for each subclass. *Different from LSF diet, p <0.05. (TIF) [file pone.0170664.s003.tif]
