# Supplementary material for: Effects of a very high saturated fat diet on LDL particles in adults with atherogenic dyslipidemia: A randomized controlled trial
Source: PLoS One. 2017 Feb 6;12(2):e0170664. doi: 10.1371/journal.pone.0170664 (PMC5293238; doi:10.1371/journal.pone.0170664)
Supplement: S1 Protocol — (PDF) [file pone.0170664.s004.pdf]

Changes in LDL and HDL with increased intake of saturated fat from dairy foods in individuals with atherogenic dyslipidemia and LDL subclass pattern B

1. Background

Dietary determinants of atherogenic dyslipidemia

Atherogenic dyslipidemia is characterized by elevated triglyceride concentrations, reduced HDL cholesterol, and increased concentrations of small, dense LDL and is highly prevalent in subjects with obesity, insulin resistance, and type 2 diabetes. Subjects displaying this predominance of small, dense LDL particles are categorized as LDL subclass pattern B. Smaller peak LDL diameter and/or elevated levels of small, dense LDL have been shown to be an independent contributor to increased cardiovascular risk in several studies [1], while a large prospective study showed that levels of large LDL subclasses do not contribute to the risk of ischemic heart disease [2], an observation recently confirmed in our own unpublished data.

We and others have previously shown that the expression of pattern B is strongly influenced by both genetic predisposition and dietary macronutrient intake. Restriction in carbohydrate intake to even moderate levels significantly decreases small, dense LDL in a manner that is independent of saturated fat intake. High carbohydrate intakes, at levels currently recommended to reduce CVD risk, promote increased plasma levels of liver-derived triglyceride-rich lipoproteins (VLDL) that can give rise to small, dense LDL particles and a conversion to or exacerbation of the pattern B phenotype [1]. Recently, we have shown that both weight loss and reduction of carbohydrate intake resulted in improvements in atherogenic dyslipidemia and that the effect of carbohydrate restriction is independent of weight loss or saturated fat intake, even at saturated fat levels exceeding current dietary recommendations [3].

Effect of saturated fat on lipoprotein metabolism

It is known that increases in saturated fat intake can lead to increases in plasma LDL cholesterol when compared to monounsaturated or polyunsaturated fat. However, it is unclear that this rise in LDL significantly affects clinical outcomes, as there is inconclusive evidence that reduced dietary saturated fat intake *per se* decreases CVD risk. Several clinical trials replacing dietary saturated fatty acids with an equivalent amount of polyunsaturated fatty acids show a reduction in CVD mortality, although not all trials do (Reviewed in [4] and [5]). Reduction of saturated fat with replacement by unsaturated fatty acids also reduces levels of HDL cholesterol and in particular, the more protective HDL2 subclass [6]. Reduction of saturated fat by replacement with carbohydrate reduces plasma LDL levels but also reduces plasma HDL, and again, mainly the HDL2 subclass [7], so that the LDL:HDL ratio is unchanged. The exchange of saturated fat for carbohydrate also increases plasma triglyceride levels, thus promoting atherogenic dyslipidemia [8]. Clinical trials reducing saturated fat by decreasing total fat and increasing carbohydrate intake have so far shown no CVD risk benefit [9]. A meta-analysis of fat intake and cardiovascular disease showed little effect on total mortality and inconclusive evidence for the effect of saturated fat on CVD events [5]. Prospective cohort studies are similarly indeterminate. In the Nurses Health Study, polyunsaturated fat was inversely and *trans* fat was positively associated with

increased risk of coronary heart disease risk after 14 and 20 years follow up, but saturated fat was not independently associated with increased risk [10, 11]. After adjusting for lifestyle and dietary variables including mono- and polyunsaturated fat, no effects of high saturated fat intake on CVD mortality was found in the Malmö Diet and Cancer study [12].

As described further below, we have observed that saturated fat from dairy sources results in selective increases in larger LDL particles that have not been related to increased CVD risk. Hence, the inconclusive evidence that saturated fat increases CVD risk is consistent with the hypothesis that the saturated fat induced rise in LDL represents increases in less atherogenic large LDL particles coupled to an increase in anti-atherogenic large HDL2 particles.

Not all saturated fatty acids similarly raise cholesterol levels. Lauric (12:0), myristic (14:0), and palmitic (16:0) acid are the major saturated fatty acids associated with an increase in plasma cholesterol levels, with myristic acid being considered the most potent of the three [13]. Epidemiological studies show a correlation of myristic acid (or myristic-lauric) intake and plasma cholesterol and prospective studies consistently show a greater cholesterol raising effect of myristic or myristic-lauric acids [13-15] when compared to palmitic acid. Myristic acid is abundant in dairy fat in the *sn*-2 position where it is found with high levels of palmitic acid. Coconut and palm kernel oils are high in myristic acid in the *sn*-1 and *sn*-3 position, although the effects of myristic acid at various positions within the triglyceride molecule are unknown.

Saturated fatty acids have been reported to decrease LDL receptor expression and activity [16, 17], thereby decreasing clearance of LDL particles from circulation. They may also regulate hepatic lipoprotein production. In rat hepatoma cells, myristic acid was found to reduce triglyceride secretion and impair the recruitment of newly synthesized triglyceride to lipoproteins [18]. This is hypothesized to lead to increased hepatic secretion of small VLDL particles that are thought to give rise to large, buoyant LDL [1]. It is also plausible that dietary saturated fat influences a common pathway that results in both increases of large LDL and large HDL. One potential mechanism is through the inhibition or reduction of cholesterol ester transfer protein (CETP). CETP mediates the transfer of cholesterol ester from HDL to apoB-containing lipoproteins in exchange for triglycerides and can modulate both size and composition of HDL and LDL. A common polymorphism in the CETP gene has been linked to decreased CETP activity, increased large HDL, larger LDL, and decreased CAD risk [19, 20]. In a population of Ashkenazi Jews, this polymorphism and the associated large HDL, large LDL phenotype was associated with exceptional longevity [19]. We have also recently obtained data indicating that several other CETP polymorphisms are very strongly associated with increased levels of larger LDL as well as HDL cholesterol. Although genetic studies suggest a benefit of reduced CETP on CVD risk, it remains to be determined whether therapeutic inhibition of CETP will reduce CVD risk. Published studies examining the effect of saturated fats on CETP are inconsistent [21-25] and it is unclear whether changes in CETP are responsible for saturated fat alterations in lipoprotein metabolism. Most studies were not designed to isolate the effects of chronic intakes of saturated fat or did not have large enough differences in saturated fat content to induce coordinate increases in both HDL and LDL. One dietary intervention study isolated the effects of saturated fat and unexpectedly saw an increase in CETP mass

(no activity was measured) in the saturated fat diet, but no increase in HDL [26]. However, this study was conducted on a background of a 47% carbohydrate diet and it may be that carbohydrate content modifies the effects of saturated fat. Our previous studies observing increases in HDL and large LDL were performed on a background of a lower carbohydrate intake. Other possible mechanisms that can result in increases in both large HDL and LDL include a reduction in hepatic lipase (HL) activity or an increase in lipoprotein lipase (LPL) activity, and again there is a lack of evidence regarding the effects of saturated fat on these enzyme activities.

We and others have consistently shown that decreases in dietary carbohydrate intake to levels below that of the typical American diet can reduce levels of LDL cholesterol, independent of body weight, even when accompanied by high intakes of saturated fat. This reduction in LDL cholesterol is specifically due to a decrease in small, dense, LDL particles in a manner that is independent of saturated fat intake (**Figure 1** and [3]). We recently demonstrated that a higher (15%) vs. lower (9%) saturated fat content in diets containing 26% carbohydrate and 29% protein increased both HDL cholesterol and total LDL mass and that this was associated with a non-significant increase in large LDL without a change in small LDL. Furthermore, there were no effects of increased saturated fat intake on apoB levels, indicating a change in composition, but not in total number of LDL particles.

Figure 1. Left panel: Changes in mass of total LDL and LDL subfractions (large=LDL I, medium=LDL II, and small=LDL III+IV) with reduced carbohydrate intake in 178 overweight and obese men. Right panel: Changes in HDL cholesterol. Changes are in reference to the baseline diet (54% carbohydrate, 16% protein, 30% fat, 7% saturated, 13% monounsaturated). After one week on the baseline diet subjects were randomized to 4 weeks of the baseline diet, or diets with reduced content of carbohydrate (39% or 26% together with increased protein (29%)). For the lowest carbohydrate intake (26%), there was also an increase in fat intake (45%), achieved either with low saturated fat (9% saturated, 27% monounsaturated or high saturated fat (15% saturated, 20% monounsaturated). Body weight was constant.

In an earlier study of the effects of macronutrient content on lipid and lipoprotein metabolism [27], we made similar observations regarding saturated fat intake and levels of large LDL. A diet with 39% carbohydrate and 46% fat resulted in higher levels of both large HDL (HDL2b) and large LDL (LDL I) compared to a low-fat (59% carbohydrate, 24% fat) diet. Based on data from diet records, the increase in large LDL was correlated with change in intake of saturated fat, specifically myristic and palmitic acids [27]. Consequently, changes in these two fatty acids were positively correlated with LDL diameter. In pattern B individuals, saturated fat intake was positively correlated with HDL2b levels. There was a significant correlation between the increase in large LDL and large HDL, suggesting that saturated fat may be acting through a common

mechanism. Intakes of stearic acid, monounsaturated and polyunsaturated fatty acids were not correlated with lipoprotein levels. The observed decrease in small LDL on the higher fat diet was likely due to a decrease in carbohydrate intake as further evidenced by subsequent studies [3, 28].

Although the results from these studies support our hypotheses, there remain gaps that this proposal will address. Our previous study testing the effects of increased saturated fat (9% vs. 15%) independent of carbohydrate intake (fixed at 26%) was not adequately powered to detect significant changes in large LDL nor did we measure HDL2b levels. In order to achieve adequate statistical power with a moderate size study population, we will use a 2-fold larger difference in dietary saturated fat content. In addition, this proposal will address the effects of saturated fat in the context of more moderate carbohydrate restriction (35%). We will test CETP, HL, and LPL as potential pathways that underlie the coordinate increases in large HDL and LDL. The proposed study will also allow for the direct testing of saturated fat effects on HDL and LDL subclass distribution in subjects with predominantly small, dense LDL. Furthermore, previous correlation studies described above utilized analytical ultracentrifugation, a method no longer in use. For this study, we will utilize our ion mobility procedure for lipoprotein subfraction analysis, an improved method that has been recently validated [29]. A representative ion mobility profile is presented in **Figure 2**.

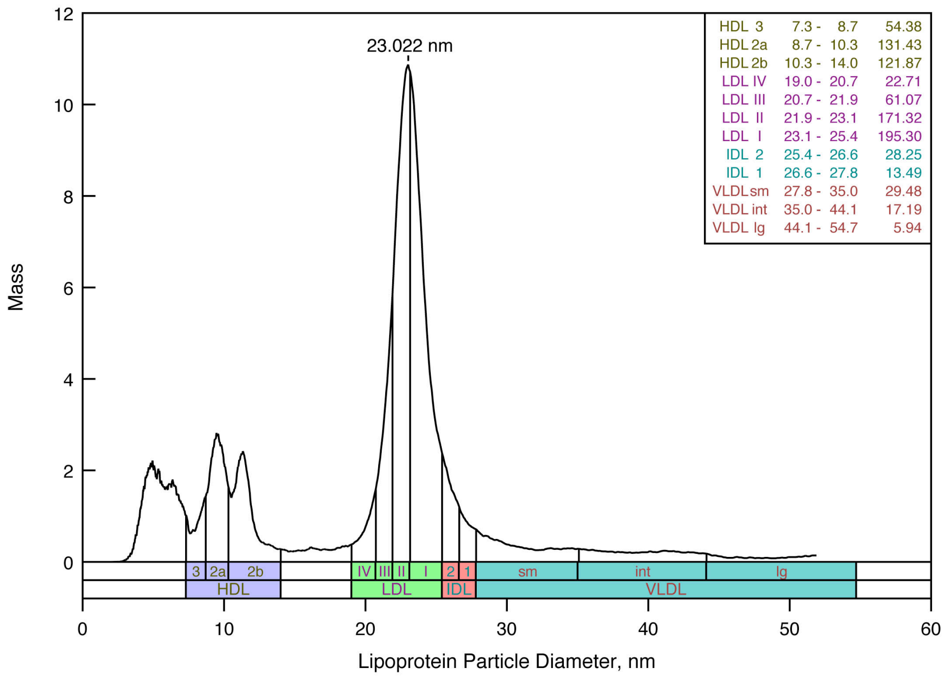

Figure 2. Representative ion mobility profile.

2. Research Project Objectives and Hypothesis

Our overall objective is to investigate the role of dairy fat in modulating lipoprotein profiles associated with cardiovascular disease (CVD) risk. In this study, we will test the following specific hypotheses regarding changes in plasma lipoproteins induced by a diet high in myristic acid and other saturated fats derived primarily from dairy sources in individuals with LDL subclass pattern B (a trait characterized by a predominance of

small, dense LDL and associated with increased CVD risk): 1) there will be a coordinate increase in levels of large HDL particles that are considered anti-atherogenic and large LDL particles that are not associated with increased CVD risk; 2) this change in lipoprotein profile will result in a higher proportion of pattern B individuals converting to LDL subclass pattern A (predominance of larger LDL); and 3) the coordinate increase in large HDL and LDL is due to a common underlying pathway. The latter hypothesis will be addressed by assaying cholesterol ester transfer protein (CETP), hepatic lipase (HL), and lipoprotein lipase (LPL).

These hypotheses will be tested by a dietary intervention study in which saturated fat intake will be increased from 8% to 20% total energy (%E) using primarily dairy products. Saturated fat will be exchanged for monounsaturated fat without changes in carbohydrate, protein, and total fat content. Furthermore, the two experimental diets will be designed to contain a difference of approximately 2.5%E of myristic acid (14:0) and 5.5%E of palmitic acid (16:0) as they are considered major cholesterol-raising fatty acids and are relatively abundant in dairy fat.

We will carry out this intervention in a controlled randomized parallel-arm study in 50 men and postmenopausal women with LDL pattern B at baseline. Participants will consume a 3-week run-in diet similar to the AHA population diet, with 55% carbohydrate, 15% protein, and 30% fat including 8% saturated and 12% monounsaturated fatty acids. They will then be randomly assigned to one of two moderate carbohydrate diets containing 35% carbohydrate, 25% protein, and 40% fat with either 8% (low saturated fat) or 20% saturated fat (high saturated fat) for 3 weeks. Following each of the two dietary periods, measurements will be made of plasma lipids, lipoproteins, lipoprotein subfractions, apolipoproteins B and AI, CETP, HL, and LPL and archived plasma, DNA, and white blood cells will be stored for future ancillary studies.

3. Project Design

Overview

The study is designed to test the hypothesis that an increase in dairy-derived saturated fat intake, in the background of a moderately reduced carbohydrate level and independent of body weight, will increase levels of large HDL and large LDL in pattern B individuals. This will be a controlled, randomized parallel-arm 6-week dietary intervention trial in 50 overweight or obese pattern B individuals. It will begin with a 3-week diet run-in period on a diet containing 55% carbohydrate, 15% protein, and 30% fat, with 8% saturated and 12% monounsaturated fat. A 3-week diet period has been shown to be sufficient for the

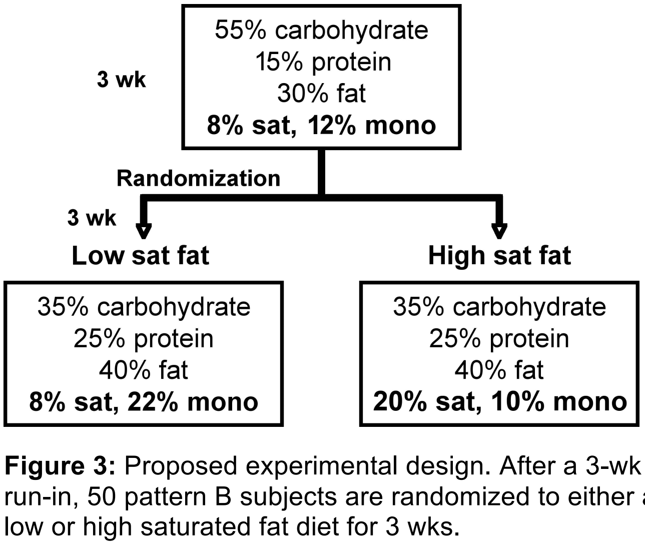

Figure 3: Proposed experimental design. After a 3-wk run-in, 50 pattern B subjects are randomized to either a low or high saturated fat diet for 3 wks.

stabilization of lipid and lipoprotein levels [3, 30]. At the end of this diet phase, subjects will be randomized to either a moderate carbohydrate, low saturated fat diet or to a moderate carbohydrate, high saturated fat diet containing the same proportions of carbohydrate (35%), protein (25%), and total fat (40%), and with either 8% or 20% saturated fat and 22% or 10% monounsaturated fat. Defining LDL pattern based on the run-in diet will allow for the subsequent comparison of pattern B to pattern A conversion rates between the high and low saturated fat diets. The expected 20% of subjects that convert to pattern A on the run-in diet will remain in the study. The two experimental diets will be designed to differ in myristic acid content by approximately 2.5% total energy and palmitic acid of approximately 5.5% (see power calculations). Based on our previous studies, these differences should be sufficient to result in selective increases in levels of large LDL particles. Dietary content of cholesterol and fiber will be similar in the two diets. Subjects will remain weight stable throughout the study. At the end of each diet phase, subjects will undergo a fasting blood draw on two consecutive days. A summary of the experimental design is shown in **Figure 3**.

<u>Diet composition</u>

Participants will be provided with two frozen entrees per day (lunch and dinner) and will prepare their meals and snacks according to the study menu. For both diets, cholesterol content will be <300 mg/day, trans fat will be <2% energy, and fiber will be 25 gm for the first 2000 kcal and 2.5 gm/500 kcal additional. Animal:vegetable protein will be held at a ~4:1 ratio on all diets. Whey protein will be incorporated into diets via milk shakes or yogurt to reach the protein requirements of the menus Diets will be low in simple sugar and high glycemic foods with a ~1:4 ratio of high glycemic index foods to low glycemic index foods. The low saturated fat diet will contain low fat/nonfat dairy while the high saturated fat diet will contain full fat dairy products. In the high saturated fat diet, dairy products will be the major source of myristic and palmitic acid in the diet. The number of dairy servings in the diets will be determined by the desired amount of total saturated fat, myristic and palmitic acids. We will develop a 4-day rotating menu for 2500, 3000, and 3500kcal levels with the addition of 250kcal snacks meeting the nutritional needs of the diet to achieve additional calorie levels. The menu and nutritional composition of a 1-day sample diet is attached (see Appendix). The average daily nutrient value of the 4-day menu is expected to reach target values described above.

Frozen entrees and menus will be developed in collaboration with the Bionutrition unit of the UCSF Clinical and Translational Studies Institute (CTSI), using a metabolic kitchen to develop custom frozen entrees. Efforts will be made to standardize ingredients and frozen entrees as fatty acid content of dairy products is known to vary somewhat based on factors such as breed, feed, and seasonal changes [31]. Custom menus and frozen entrees will be created using ProNutra software (Viocare Technologies, Inc) and a composite of each daily menu will be analyzed for nutritional composition through Covance Laboratories, Inc.

<u>Participant Recruitment and Selection</u>

We will recruit subjects from an extensive database generated from previous clinical studies and through the community at large through bulk mailing, outreach

programs, posters, radio, newspaper, and internet advertisements. A screening questionnaire and blood draw will determine eligibility based on the following criteria:

1) Men or postmenopausal women (>6 months since last menses) between the ages of 18 and 70.
2) No history of coronary heart disease, cerebrovascular disease, peripheral vascular disease, bleeding disorder, liver or renal disease, diabetes, lung disease, HIV, or cancer (other than skin cancer) in the last 5 years.
3) Not taking drugs known to affect lipid metabolism or hormones
4) Blood pressure less than 150/90. If on three separate clinic visits blood pressure remains above this level, a subject will be referred to his or her physician for treatment.
5) Body mass index (BMI) ≥ 25 and ≤ 35 kg/m^2.
6) Non-smoking.
7) Agrees to consume no alcohol or dietary supplements during the study.
8) Total cholesterol and LDL cholesterol ≤95th percentile for sex and age.
9) Fasting triglycerides ≤500mg/dl.
10) Pattern B (LDL peak particle size < 208.8Å by ion mobility).
11) Fasting blood sugar (FBS) < 126 mg/dl.
12) At least 3 months of a weight stable state. During the study, subjects will be required to maintain their body weight within ± 3% (up to a maximum change of 5 lbs) of their initial weight over the course of any consecutive two weeks.
13) Normal thyroid stimulating hormone (TSH) levels.
14) Ability to follow the study protocol. If a potential participant cannot complete the requirements of the study for reasons determined by the investigator (i.e. non-accessible veins for blood drawing, inability to keep clinic appointments), they will be considered ineligible. If they are already enrolled in the study, they will be withdrawn.

As an incentive for participation, subjects will be offered an optional four weeks of weight loss counseling with a registered dietitian who will provide a reduced-calorie exchange diet, written materials on weight management, and physical activity program of at least 7,500 steps/day. The participant will visit the dietitian weekly. If the subject chooses, a blood draw (15ml) will be taken at the end of the four weeks for lipid and lipoprotein analysis. Participants completing the study will be reimbursed $750 for food purchases.

Based on data from previous dietary studies, we expect approximately 50% of those participants screened to be pattern B. Therefore, using a conservative estimate of 40% of screened subjects qualifying as pattern B, for 50 completed subjects and assuming 20% of subjects decline participation after screening and a 20% drop out rate, we will need to screen approximately 200 subjects and enroll 64 subjects. Recruitment will be facilitated by concurrent efforts to recruit for our current dietary study sponsored by Dairy Management, Inc. The recruitment and enrollment schedule is outlined in **Figure 4.**

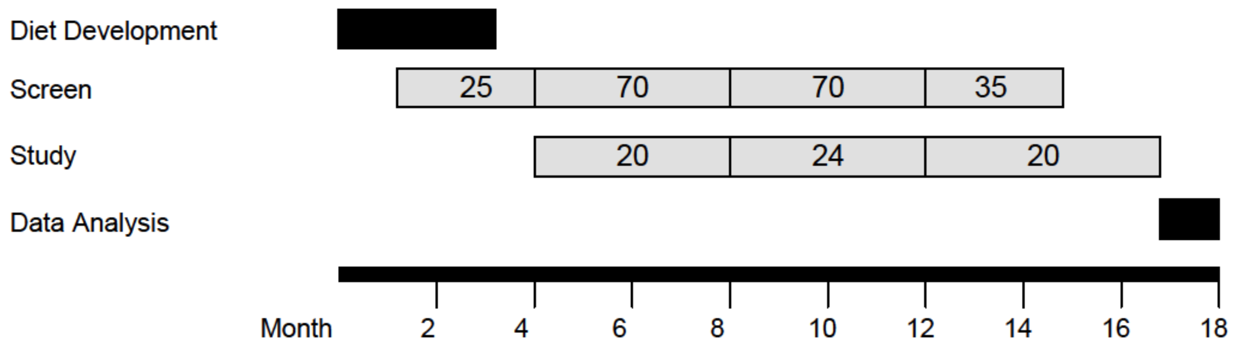

Figure 4: Study schedule. Participant numbers given are based on 64 participants randomized to account for a 20% drop out rate. To account for the prevalence of pattern B subjects in this population, and subjects that decline participation after screening, 200 participants will need to be screened.

4. Research Methods

Clinical protocol

A. Overview

Similar to our previous work, this study will be conducted as an outpatient study with careful monitoring of dietary compliance. We will use conventional food diets, providing participants with two frozen entrees per day and a menu checklist of additional items to be prepared and consumed as instructed. Throughout the study, subjects will wear a pedometer and be instructed to target their average steps/day as determined by baseline assessment (week 1 of baseline diet) of their activity level. Subjects will remain weight stable (± 3%, up to 5lbs.) throughout the study.

Dietary compliance will be facilitated by 1) provision of standardized menus with shopping lists and written instructions, 2) menus with checklists for recording daily food intake, adherence, and deviations, 3) consultation at weekly dietitian visits, 4) weekly weight measurement and diet review, 5) collection of store receipts to document purchase of menu items. We and others [33, 34] have found that follow-up counseling after the initial education on diet has been an effective compliance tool. The majority of participants are able to make appropriate adaptations during short-term diet therapy [35, 36].

B. Clinic Visits

Including the screening visit, subjects will visit the clinic for a total of 6 blood draws and will meet with the dietitian on 6 different occasions. A summary of the clinic visits is shown in **Figure 5**.

<u>Screening Visit (V1):</u> Interested subjects eligible through a screening questionnaire and telephone conversation will be mailed an orientation packet followed by telephone consultation with the dietitian. At the screening visit, subjects will give informed consent, have their medical history reviewed, blood pressure taken, and anthropometrics measured. Anthropometric measurements include weight, height, waist and hip

circumference, and % body fat by bioimpedance scale (Tanita). Subjects will give a fasting blood draw to determine final eligibility.

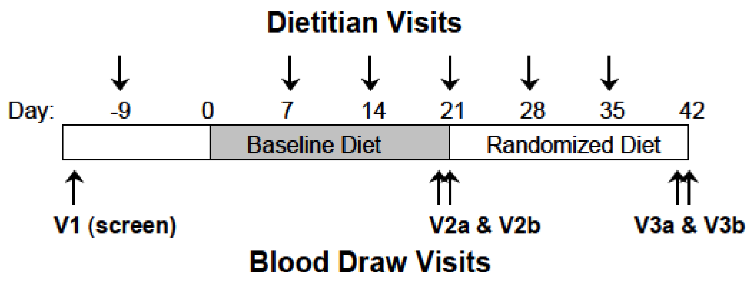

Figure 5. Clinic visit schedule.

Dietitian Visits: Upon entry to the study, subjects will visit the clinic for the first dietitian visit where they will receive detailed dietary instructions and consultation. Subjects will be provided with written instructions, a food scale, shopping lists, menu checklists and frozen entrees for the week. Subjects will be weighed and will take a food frequency questionnaire (Block 2005.1) to assess their usual diet. Subsequent to the first dietitian visit, subjects will meet with the dietitian weekly to be weighed, turn in their menu checklists, receipts, and activity logs, and pick up their menus and frozen entrees.

Randomization Visits (V2a & V2b): After completing the run-in dietary period, subjects will visit the clinic on two subsequent days (within 24-96 hrs apart). At V2a, subjects will have their blood pressure, body weight, waist and hip circumference, and % body fat (Tanita) measured. The will undergo a fasting and post-heparin blood draw. The following day (V2b), subjects will visit the clinic for a fasting blood draw. The purpose of the V2b visit is to increase the statistical power of analyses. Subjects will also have their weekly dietitian visit at V2b.

Completion Visits (V3a & V3b): After completing the randomized diet phase, subjects will visit the clinic on two subsequent days (within 24-96 hrs apart). At V3a, subjects will have their blood pressure, body weight, waist and hip circumference, and % body fat (Tanita) measured and will undergo a fasting and post-heparin blood draw. The following day (V3b), subjects will visit the clinic for a fasting blood draw to increase statistical power.

Clinical Procedures

Clinical measurements: Blood pressure will be measured 3 times in a sitting position and the last 2 values averaged. Anthropometric measurements include height, weight, waist and hip circumference, and % body fat by bioimpedance (Tanita scale). In our previous study (P. Siri Tarino, unpublished), we showed that the major results using this measurement did not differ significantly from those by DXA. Waist circumference is measured two times at the iliac crest and hip circumference is measured at the widest point of the hips.

Blood sampling: Using standard blood collection procedures, blood samples will be collected from participants after a 12-14 hour fast. The blood will be collected into tubes containing the following preservative solution: 3.0 gms EDTA (dipotassium), 1.7 mg P-Pack, 0.15 gms gentamycin sulfate, 0.15 gms chloramphenicol, 5.96 mls aprotinin (Sigma A-6279), and 0.30 gms sodium azide all of which are diluted to 20mls with

doubly deionized water. Plasma is separated by immediate centrifugation at 4°C. Lipid and lipoprotein measurements are performed as described below and aliquots of plasma are frozen for future analyses (8, 1ml each). Post-heparin plasma samples will be collected after a heparin bolus for lipase activity assays.

Plasma analyses: At the screening visit (V1), plasma will be analyzed for total cholesterol, LDL-cholesterol, HDL-cholesterol, triglycerides, glucose, TSH, and LDL peak particle size for LDL phenotype determination. At V2a and V3a (first visits before and after randomized diet), measurements will include total cholesterol, LDL-cholesterol, HDL-cholesterol, triglycerides, apolipoprotein A1 and B, LDL phenotyping, lipoprotein subfraction analysis by ion mobility, CETP activity, and post-heparin HL and LPL activity. We will also collect white blood cells and buffy coats for future genetic analyses. A portion of the white blood cells will be immortalized to generate permanent cell lines. At visits V2b and V3b (second visits before and after randomized diet), measurements will include total cholesterol, LDL-cholesterol, HDL-cholesterol, triglycerides, and lipoprotein subfraction analysis by ion mobility. The blood-drawing schedule is shown in **Figure 5.**

Adverse event reporting. At each blood draw visit, a registered nurse will discuss with the participant any adverse events that may have occurred and will note any events in the visit record. A registered nurse will also be available by phone to discuss any events that occur during the study. All non-serious events will be documented in an event tracking log, individually reviewed by the PI, and reported to the IRB per policy. Serious or unexpected adverse events will be reported per IRB policy and will be documented in the participant's chart and database record.

Laboratory procedures

Lipid analyses. Human plasma will be analyzed for concentrations of total cholesterol [37] and triglyceride [38], and for high density lipoprotein (HDL)-cholesterol, measured directly after precipitation of apolipoprotein B containing lipoproteins in plasma [39]. LDL cholesterol is calculated by subtraction of estimated VLDL and measured HDL cholesterol from the measured total cholesterol and triglyceride in plasma. Lipid assays are enzymatic end-point measurements utilizing enzyme reagent kits (Ciba-Corning Diagnostics Corp., Oberlin, Ohio) and a CIBA Corning Express 550 automated analyzer. The measurements are standardized through the CDC-NHLBI Lipid Standardization Program.

Apolipoprotein B and AI : Immunoturbidimetric assay [40, 41] will be used to measure apolipoproteins AI and B in human plasma samples. Reagents, standards, and reference plasma controls, with and without elevated lipids, are included in the ITA reagent kit (Bacton Assay Systems, Inc., San Marcos, CA). Measurements will be performed using the Express Plus 550 analyzer. Calibrators and reference controls are assigned concentration values with the use of International Federation of Clinical Chemistry standard reference materials SP1 for apoAI and SP3-07 for apoB.

Lipoprotein subclass analysis by ion mobility (IM). LDL and HDL subfractions will be analyzed using our in-house method, ion mobility (IM), which uniquely allows for direct particle quantification following an ultracentrifugation to remove albumin. The IM instrument utilizes an electrospray to create an aerosol of particles, which then pass

through a dynamic mobility analyzer (DMA) coupled to a particle counter [29]. Particle diameter and quantity are determined and converted to lipoprotein mass and LDL peak particle size. Interassay variation is reduced by inclusion of two in-house controls in each preparatory process.

Hepatic and lipoprotein lipase activity. Total hepatic (HL) and lipoprotein lipase (LPL) activity will be measured in post-heparin plasma samples by incubation with a standard concentration of isotope-labeled esterified fatty acids (glycerol tri[^{3}H]-oleate) followed by quantification of extracted free fatty acids using a liquid scintillation counter [42]. HL activity is determined using selective inhibition with protamine sulfate. LPL activity will be calculated as the difference between total post-heparin lipase activity and HL activity.

Cholesterol ester transfer protein (CETP) activity. CETP activity will be measured by a commercially available activity assay kit (Roar Biomedical, Inc) according to manufacturer's recommendations [43, 44]. In-house frozen control plasmas will be used to monitor assay variability.

Cholesterol ester transfer protein (CETP) mass. Plasma concentrations of CETP protein will be measured by ELISA using a commercially available kit (Alpco Diagnostics) according to kit instructions. In-house frozen control plasmas will be used to monitor assay variability.

Plasma glucose. Glucose will by measured using enzymatic reagent kits (Glucose HK Reagent, Ciba Corning Diagnostics) and interassay variation will be monitored by using in-house frozen control plasmas.

Thyroid stimulating hormone. Plasma thyroid stimulating hormone (TSH) will be sent to Quest Diagnostics for analysis.

Data Analysis

The hypothesis that increased saturated fat derived primarily from dairy products will increase levels of large HDL2 and large LDL in pattern B individuals, with no significant effect on small, dense LDL, will be tested by comparing the mean change in large LDL levels as measured by ion mobility. The statistical significance will be evaluated by one-way analysis of variance with the factor being high or low saturated fat intake. Similar analyses will be used to test the effects of saturated fat on HDL cholesterol, and LDL diameter. Comparison of LDL pattern conversion rate between high and low saturated fat diets will be analyzed by Chi-squared analysis.

Table 1 shows the detectable and expected differences using data from the high fat/low fat study described above [27]. Expected differences are derived from correlations of the change in fatty acid intake (assessed by food records of prescribed diets) and change in LDL measurements. The correlations were used to select the differences in SFA (12%), myristic (2.5%), and palmitic acid (5.5%) levels needed for a detectable effect in the primary endpoints given 25 subjects per group. These are approximately equal to the mean change in intake in our previous study. With 25 subjects/group, we have adequate power (80%) to detect the expected changes in LDL I, LDL diameter, and HDL2b given either a 12% difference in saturated fat or a 2.5% difference in myristic acid between the two diets. Although the lipoprotein subfraction predictions are based on measurements made by analytical ultracentrifugation or gradient gel electrophoresis, we expect the ion mobility method to be equally or more

precise. Typical standard deviations for measurements by ion mobility are 20.9 mg/dl for large LDL and 3.5 Å for LDL diameter. In addition, we will use the average value of measurements from two blood draws on consecutive days to reduce variance and increase power.

Table 1: Detectable (80% power) and expected difference for primary endpoints

	SD of difference	Detectable differences between groups[1]		Expected Difference w/ 12% ΔSFA	Expected Difference w/ 2.5% Δ14:0
		Difference	% baseline		
HDL2b (mg/dl)	11.5	6.5	25	7.9 mg/dl	10.7 mg/dl
LDL-I (mg/dl)	29.6	16.6	22	39.2 mg/dl	49.6 mg/dl
LDL diam (Å)	5.0	2.9	1.7	4.9 Å	6.1 Å

[1]Detectable differences assume the average of 2 baseline and 2 post-diet measurements

To estimate predicted LDL pattern conversion rates, we used logisitic regression to achieve a best approximation of the expected conversion rate between the two diets. At 35% carbohydrate intake and 12% difference in saturated fat, we expect that 48% pattern B participants will convert to pattern A. With a 2.5% difference in myristic acid, we predict a 71% conversion rate. Similar power estimates were obtained with a 5.5% difference in palmitic acid.

Other endpoints will include plasma triglyceride, total cholesterol, LDL-cholesterol, HDL cholesterol, apoB, apoAI, and LDLII-IV. **Table 2** presents the detectable differences in these endpoints estimated at 80% statistical power, 5% significance using baseline concentrations and standard deviation of response observed in our previous controlled dietary trials. Detectable differences are in accordance with power estimates from previous diet studies.

Sample size estimation is determined by lipoprotein endpoints, as not enough data is available to estimate expected differences in CETP, HL, and LPL activity levels. Based on previous data, we have adequate power (86%) to detect a difference of 15% baseline levels of HL activity between groups and reasonable power (52%) to detect a difference of 30% baseline levels of LPL between groups.

Table 2. Detectable differences (80% power) in other plasma lipid and lipoprotein levels

	Baseline concentrations	Standard deviation of difference	Differences between groups	
			Detectable Difference[1]	% baseline
Triglycerides (mg/dL)	215.31±106.34	117.97	65.98	30.65
Total cholesterol (mg/dL)	206.79±32.53	25.97	14.52	7.02
LDL-chol(mg/dL)	127.55±30.19	24.60	13.76	10.79
HDL-C(mg/dl)	36.49±6.05	5.34	3.05	8.36
Apo AI (mg/dL)	109.21±14.17	9.49	5.31	4.87
Apo B (mg/dL)	99.78±18.03	25.50	14.26	14.29
LDL-2 (mg/dL)[2]	90.03±38.62	35.00	19.58	21.75
LDL-3 (mg/dL)[2]	91.33±28.76	25.02	13.99	15.31
LDL-4 (mg/dL)[2]	18.72±14.39	15.71	8.78	46.92

[1]Detectable differences assume the average of 2 baseline and 2 post-diet measurements
[2]LDL subfraction data for this table was derived from a more recent study than data shown in Table 1, but show very similar % baseline differences.

References

1. Berneis, K.K. and R.M. Krauss, *Metabolic origins and clinical significance of LDL heterogeneity.* J Lipid Res, 2002. **43**(9): p. 1363-79.
2. St-Pierre, A.C., et al., *Low-density lipoprotein subfractions and the long-term risk of ischemic heart disease in men: 13-year follow-up data from the Quebec Cardiovascular Study.* Arterioscler Thromb Vasc Biol, 2005. **25**(3): p. 553-9.
3. Krauss, R.M., et al., *Separate effects of reduced carbohydrate intake and weight loss on atherogenic dyslipidemia.* Am J Clin Nutr, 2006. **83**(5): p. 1025-31; quiz 1205.
4. Hu, F.B. and W.C. Willett, *Optimal diets for prevention of coronary heart disease.* JAMA, 2002. **288**(20): p. 2569-78.
5. Sacks, F.M. and M. Katan, *Randomized clinical trials on the effects of dietary fat and carbohydrate on plasma lipoproteins and cardiovascular disease.* Am J Med, 2002. **113 Suppl 9B**: p. 13S-24S.
6. Lichtenstein, A.H., et al., *Impact of hydrogenated fat on high density lipoprotein subfractions and metabolism.* J Lipid Res, 2001. **42**(4): p. 597-604.
7. Berglund, L., et al., *HDL-subpopulation patterns in response to reductions in dietary total and saturated fat intakes in healthy subjects.* Am J Clin Nutr, 1999. **70**(6): p. 992-1000.
8. Siri, P.W. and R.M. Krauss, *Influence of dietary carbohydrate and fat on LDL and HDL particle distributions.* Curr Atheroscler Rep, 2005. **7**(6): p. 455-9.
9. Burr, M.L., et al., *Effects of changes in fat, fish, and fibre intakes on death and myocardial reinfarction: diet and reinfarction trial (DART).* Lancet, 1989. **2**(8666): p. 757-61.
10. Hu, F.B., et al., *Dietary fat intake and the risk of coronary heart disease in women.* N Engl J Med, 1997. **337**(21): p. 1491-9.
11. Oh, K., et al., *Dietary fat intake and risk of coronary heart disease in women: 20 years of follow-up of the nurses' health study.* Am J Epidemiol, 2005. **161**(7): p. 672-9.
12. Leosdottir, M., et al., *Dietary fat intake and early mortality patterns--data from The Malmo Diet and Cancer Study.* J Intern Med, 2005. **258**(2): p. 153-65.
13. Zock, P.L., J.H. de Vries, and M.B. Katan, *Impact of myristic acid versus palmitic acid on serum lipid and lipoprotein levels in healthy women and men.* Arterioscler Thromb, 1994. **14**(4): p. 567-75.
14. Khosla, P., et al., *Decreasing dietary lauric and myristic acids improves plasma lipids more favorably than decreasing dietary palmitic acid in rhesus monkeys fed AHA step 1 type diets.* J Nutr, 1997. **127**(3): p. 525S-530S.
15. Sundram, K., K.C. Hayes, and O.H. Siru, *Dietary palmitic acid results in lower serum cholesterol than does a lauric-myristic acid combination in normolipemic humans.* Am J Clin Nutr, 1994. **59**(4): p. 841-6.

16. Woollett, L.A., D.K. Spady, and J.M. Dietschy, *Saturated and unsaturated fatty acids independently regulate low density lipoprotein receptor activity and production rate.* J Lipid Res, 1992. **33**(1): p. 77-88.
17. Bennett, A.J., et al., *Modulation of hepatic apolipoprotein B, 3-hydroxy-3-methylglutaryl-CoA reductase and low-density lipoprotein receptor mRNA and plasma lipoprotein concentrations by defined dietary fats. Comparison of trimyristin, tripalmitin, tristearin and triolein.* Biochem J, 1995. **311 (Pt 1)**: p. 167-73.
18. Kummrow, E., et al., *Myristic acid increases dense lipoprotein secretion by inhibiting apoB degradation and triglyceride recruitment.* J Lipid Res, 2002. **43**(12): p. 2155-63.
19. Barzilai, N., et al., *Unique lipoprotein phenotype and genotype associated with exceptional longevity.* JAMA, 2003. **290**(15): p. 2030-40.
20. Thompson, A., et al., *Association of cholesteryl ester transfer protein genotypes with CETP mass and activity, lipid levels, and coronary risk.* JAMA, 2008. **299**(23): p. 2777-88.
21. Cheema, S.K., et al., *Lack of stimulation of cholesteryl ester transfer protein by cholesterol in the presence of a high-fat diet.* J Lipid Res, 2005. **46**(11): p. 2356-66.
22. Chung, B.H., et al., *Contribution of postprandial lipemia to the dietary fat-mediated changes in endogenous lipoprotein-cholesterol concentrations in humans.* Am J Clin Nutr, 2004. **80**(5): p. 1145-58.
23. Fielding, C.J., et al., *Effects of dietary cholesterol and fat saturation on plasma lipoproteins in an ethnically diverse population of healthy young men.* J Clin Invest, 1995. **95**(2): p. 611-8.
24. Fusegawa, Y., et al., *Influence of dietary fatty acid composition on the relationship between CETP activity and plasma lipoproteins in monkeys.* J Lipid Res, 2001. **42**(11): p. 1849-57.
25. Gupta, S.V., et al., *Replacing 40% of dietary animal fat with vegetable oil is associated with lower HDL cholesterol and higher cholesterol ester transfer protein in cynomolgus monkeys fed sufficient linoleic acid.* J Nutr, 2003. **133**(8): p. 2600-6.
26. Jansen, S., et al., *Low-fat and high-monounsaturated fatty acid diets decrease plasma cholesterol ester transfer protein concentrations in young, healthy, normolipemic men.* Am J Clin Nutr, 2000. **72**(1): p. 36-41.
27. Dreon, D.M., et al., *Change in dietary saturated fat intake is correlated with change in mass of large low-density-lipoprotein particles in men.* Am J Clin Nutr, 1998. **67**(5): p. 828-36.
28. Dreon, D.M., et al., *A very low-fat diet is not associated with improved lipoprotein profiles in men with a predominance of large, low-density lipoproteins.* Am J Clin Nutr, 1999. **69**(3): p. 411-8.
29. Caulfield, M.P., et al., *Direct Determination of Lipoprotein Particle Sizes and Concentrations by Ion Mobility Analysis.* Clin Chem, 2008.
30. Kris-Etherton, P.M., et al., *High-monounsaturated fatty acid diets lower both plasma cholesterol and triacylglycerol concentrations.* Am J Clin Nutr, 1999. **70**(6): p. 1009-15.

31. Jensen, R.G., *The composition of bovine milk lipids: January 1995 to December 2000.* J Dairy Sci, 2002. **85**(2): p. 295-350.
32. McLaughlin, T., et al., *Is there a simple way to identify insulin-resistant individuals at increased risk of cardiovascular disease?* Am J Cardiol, 2005. **96**(3): p. 399-404.
33. Milkereit, J. and J.S. Graves, *Follow-up dietary counseling benefits attainment of intake goals for total fat, saturated fat, and fiber.* J Am Diet Assoc, 1992. **92**(5): p. 603-5.
34. Shenberger, D.M., et al., *Intense dietary counseling lowers LDL cholesterol in the recruitment phase of a clinical trial of men who had coronary artery bypass grafts.* J Am Diet Assoc, 1992. **92**(4): p. 441-5.
35. Ginsberg, H.N., et al., *Reduction of plasma cholesterol levels in normal men on an American Heart Association Step 1 diet or a Step 1 diet with added monounsaturated fat.* N Engl J Med, 1990. **322**(9): p. 574-9.
36. Gordon, D.J., et al., *Dietary determinants of plasma cholesterol change in the recruitment phase of the Lipid Research Clinics Coronary Primary Prevention Trial.* Arteriosclerosis, 1982. **2**(6): p. 537-48.
37. Allain, C.C., et al., *Enzymatic determination of total serum cholesterol.* Clin Chem, 1974. **20**(4): p. 470-5.
38. Nagele, U., et al., *Reagent for the enzymatic determination of serum total triglycerides with improved lipolytic efficiency.* J Clin Chem Clin Biochem, 1984. **22**(2): p. 165-74.
39. Warnick, G.R., T. Nguyen, and A.A. Albers, *Comparison of improved precipitation methods for quantification of high-density lipoprotein cholesterol.* Clin Chem, 1985. **31**(2): p. 217-22.
40. Rifai, N. and M.E. King, *Immunoturbidimetric assays of apolipoproteins A, AI, AII, and B in serum.* Clin Chem, 1986. **32**(6): p. 957-61.
41. Smith, S.J., et al., *An international collaborative study on standardization of apolipoproteins A-I and B. Part I. Evaluation of a lyophilized candidate reference and calibration material.* Clin Chem, 1987. **33**(12): p. 2240-9.
42. Campos, H., D.M. Dreon, and R.M. Krauss, *Associations of hepatic and lipoprotein lipase activities with changes in dietary composition and low density lipoprotein subclasses.* J Lipid Res, 1995. **36**(3): p. 462-72.
43. Harder, C., et al., *Cholesteryl ester transfer protein (CETP) expression protects against diet induced atherosclerosis in SR-BI deficient mice.* Arterioscler Thromb Vasc Biol, 2007. **27**(4): p. 858-64.
44. Rittershaus, C.W., et al., *Vaccine-induced antibodies inhibit CETP activity in vivo and reduce aortic lesions in a rabbit model of atherosclerosis.* Arterioscler Thromb Vasc Biol, 2000. **20**(9): p. 2106-12.
