# Supplementary material for: Effects of a very high saturated fat diet on LDL particles in adults with atherogenic dyslipidemia: A randomized controlled trial
Source: PLoS One. 2017 Feb 6;12(2):e0170664. doi: 10.1371/journal.pone.0170664 (PMC5293238; doi:10.1371/journal.pone.0170664)
Supplement: S1 Table — (PDF) [file pone.0170664.s005.pdf]

S1 Table: Spearman's rho correlations of change in enzyme activities and change in lipid and lipoproteins[1]

	Δ HL	Δ LPL	Δ CETP
ΔTG	0.35[a]	-0.34[a]	0.35[b]
ΔTC	0.32[a]	0.02	0.43[b]
ΔLDL-C	0.16	0.20	0.26
ΔHDL-C	-0.14	0.09	0.00
ΔNonHDL-C	0.35[a]	0.01	0.41[b]
ΔApoB	0.36[a]	0.01	0.41[b]
ΔApoAI	0.22	0.11	0.26
ΔTotal VLDL	0.35[a]	-0.19	0.33[a]
ΔLarge VLDL	0.40[b]	-0.36[a]	0.32[a]
ΔMedium VLDL	0.31[a]	-0.20	0.31[a]
ΔSmall VLDL	0.18	-0.14	0.29[a]
ΔIDL	0.24	0.05	0.37[b]
ΔTotal LDL	0.26	-0.12	0.40[b]
ΔLarge LDL	-0.11	0.07	0.05
ΔMedium LDL	0.15	0.03	0.31[a]
ΔSmall LDL	0.31[a]	-0.15	0.29[a]
ΔVery small LDL	0.41[b]	-0.25	0.21
ΔLDL peak diameter	-0.28[a]	0.20	-0.20
ΔTotal HDL	0.24	0.01	0.26
ΔLarge HDL	0.16	-0.06	0.16
ΔSmall HDL	0.26	0.05	0.25

[1] Difference in the log transformed values. [a] $0.05>p>0.01$; [b] $p<0.01$. Apo, apolipoprotein; CETP, cholesteryl ester transfer protein; HL, hepatic lipase; HDL-C, HDL-cholesterol; IDL, intermediate density lipoprotein; LPL, lipoprotein lipase; LDL-C, LDL cholesterol; TC, total cholesterol; TG, triglycerides; VLDL, very low density lipoprotein.
